# Operating length and velocity of human M. vastus lateralis fascicles during vertical jumping

**DOI:** 10.1098/rsos.170185

**Published:** 2017-05-03

**Authors:** Maria Elissavet Nikolaidou, Robert Marzilger, Sebastian Bohm, Falk Mersmann, Adamantios Arampatzis

**Affiliations:** 1 School of Physical Education and Sport Science, National and Kapodistrian University of Athens, Athens, Greece; 2 Department of Training and Movement Sciences, Humboldt-Universität zu Berlin, Berlin, Germany; 3 Berlin School of Movement Science, Berlin, Germany

**Keywords:** force–length–velocity potential, power–velocity potential, activation, fascicle length, fascicle-shortening velocity, joint moment

## Abstract

Humans achieve greater jump height during a counter-movement jump (CMJ) than in a squat jump (SJ). However, the crucial difference is the mean mechanical power output during the propulsion phase, which could be determined by intrinsic neuro-muscular mechanisms for power production. We measured M. vastus lateralis (VL) fascicle length changes and activation patterns and assessed the force–length, force–velocity and power–velocity potentials during the jumps. Compared with the SJ, the VL fascicles operated on a more favourable portion of the force–length curve (7% greater force potential, i.e. fraction of VL maximum force according to the force–length relationship) and more disadvantageous portion of the force–velocity curve (11% lower force potential, i.e. fraction of VL maximum force according to the force–velocity relationship) in the CMJ, indicating a reciprocal effect of force–length and force–velocity potentials for force generation. The higher muscle activation (15%) could therefore explain the moderately greater jump height (5%) in the CMJ. The mean fascicle-shortening velocity in the CMJ was closer to the plateau of the power–velocity curve, which resulted in a greater (15%) power–velocity potential (i.e. fraction of VL maximum power according to the power–velocity relationship). Our findings provide evidence for a cumulative effect of three different mechanisms—i.e. greater force–length potential, greater power–velocity potential and greater muscle activity—for an advantaged power production in the CMJ contributing to the marked difference in mean mechanical power (56%) compared with SJ.

## 1. Introduction

It is well known that humans achieve greater jump height during the so-called countermovement jump (CMJ) compared with the squat jump (SJ) [1–3], with average differences ranging from 5 to 10% [2,4]. Jump height during SJ and CMJ is determined by the total mechanical work performed by the muscle-tendon units during the propulsion phase [2,4]. This means that, given a controlled starting position in both jumps (i.e. similar length of the muscle-tendon units at the beginning of the propulsion phase), the mechanical work performed during the jump and the jump height achieved depend on the muscle forces generated in the lower extremities muscle-tendon units during the propulsion phase [2,4]. There is indeed evidence that a higher activation of the muscles during the propulsion phase and especially at the beginning of the push-off would be the responsible mechanism for the higher jumping height in CMJ [5].

The crucial difference between the two jumps is the mean mechanical power output achieved during the propulsion phase (i.e. rate of the total energy of the centre of mass). In the CMJ, the mean mechanical power output is about 47% higher compared with the SJ [6,7]. A higher muscle activation during the preparatory countermovement may increase the level as well as the development of the muscle force and in this way the transfer of elastic strain energy to the tendons [2,4]. The increased elastic strain energy in the tendons can enhance the mean mechanical power output during the propulsion phase of the CMJ in a catapult-like manner [8]. Yet muscle force and power are not only dependent on the muscle activation level but also on the force–length and force/power–velocity potentials of the muscle (i.e. fraction of maximal force and maximal power according to the force–length, force–velocity and power–velocity relationships; [9–11]). Lutz & Rome [10] provided evidence that beyond muscle activation the operating length and velocity of the sarcomeres affect muscle force and power production during jumping. They found that the semimembranosus muscle of frogs during a maximal jump was activated maximally and operated near to the optimal sarcomere length and optimal muscle shortening velocity to produce maximal power [10]. It seems possible that differences between SJ and CMJ with regard to fascicle length and fascicle-shortening velocity of the contributing muscles during the propulsion phase may influence the muscle force and power production at a given muscle activation. It has been demonstrated that elastic mechanisms, for example tendon compliance, are essential for effective locomotion in animals [12,13] and humans [14,15]. Owing to their compliance, tendons can reduce the shortening velocity of the muscles [12] and regulate the operating muscle length [16,17] in order to increase the muscle force and power potentials and might in this way also influence muscle force and power generation during jumping. The time course of activation (i.e. neural control of muscle contraction) plays a key role in the interaction between tendons and muscles and can affect the efficiency of muscle force and power production [18–20].

Kurukawa *et al*. [21,22] investigated *in vivo* the fascicle length of the gastrocnemius medialis (GM) muscle during SJ and CMJ and found a quite similar fascicle behaviour between the two conditions (i.e. the fascicles operated at similar length near to the optimum of the force–length relationship) and, therefore, at a comparable muscle force potential. In both jumps, the fascicles of the GM shortened during the whole motion without any stretch-shortening behaviour, even in the CMJ, and generated similar power [22]. Compared with the triceps surae muscles, the quadriceps femoris muscles possess a different morphology with longer fascicle length and greater muscle volume [23–25] and, therefore, are able to produce greater energy and power. Finni *et al*. [26] measured the Achilles and patellar tendon force during SJ and CMJ *in vivo* using the optical fibre methodology and reported greater peak mechanical power of the quadriceps femoris (a factor of approximately 2.5) compared with the triceps surae muscle. Musculoskeletal models predict a major contribution of the vastii muscles to mechanical energy and power production during both SJ and CMJ [4,7]. Considering that the monoarticular knee extensor muscles (i.e. vastus medialis, vastus lateralis and vastus intermedius) operate in their optimal length in a knee flexion angle of 50° to 60° [27,28], it can be argued that during SJ and CMJ at the start of the push-off phase the vastii muscles act at the descending part of their force–length curve.

Starting the contraction at longer muscle lengths (i.e. descending part of the force–length curve) and shortening onto the optimal length favours the development of muscle force and results in high peak forces during jumping [16]. Assuming that during both SJ and CMJ the monoarticular vastii muscles operate at the descending part of the force–length curve, we can expect that the higher activation of the vastii at the beginning of the push-off phase in the CMJ [2,4] results in higher muscle forces compared with SJ. Higher muscle forces of the vastii at the start of the push-off phase may shorten the fascicle length in the CMJ due to the tendon compliance, increasing the muscle force–length potential compared with SJ. Further, the duration of the push-off phase during CMJ is 20% to 30% shorter compared with SJ [2,7], which indicates differences in the shortening velocity of the monoarticular vastii muscles and, thus, effects on muscle force and power production due to the force–velocity and power–velocity relationships.

To the best of our knowledge, the fascicle behaviour, operating length and shortening velocity of the vastii muscles during SJ and CMJ *in vivo* have not been investigated yet and our current understanding of how muscle mechanics might be related to the increased performance in the CMJ compared with the SJ is still deficient. In this study, we measured the vastus lateralis (VL) fascicle length and electromyographic activity (EMG) as a representative of the monoarticular knee extensor muscles during SJ and CMJ. Further, we experimentally assessed the force–length relationship of the VL muscle using several maximal isometric contractions and calculated the maximal shortening velocity of the VL using muscle-specific parameters from literature reports [29]. The purpose of this study was to investigate the operating length and velocity of the VL muscle fascicles regarding force and power generation during the SJ and CMJ *in vivo*. We hypothesized that the fascicles of the VL operate at a different length and velocity during the two jumps and that intrinsic mechanical mechanisms (i.e. force–length and power–velocity relationships) of the VL muscle to generate force and power contribute to the substantially higher mean mechanical power output in CMJ compared with the SJ. We predicted a more favourable operating length and shortening velocity closer to the optimum for force and power generation, respectively, for the CMJ compared with the SJ.

## 2. Material and methods

### 2.1. Experimental protocol

Seventeen physically active participants (age, 27.0 ± 4.1 years; body mass, 76.8 ± 8.8 kg; height, 179.5 ± 6.2 cm) with no clinical evidence of neuro-musculoskeletal injury or abnormalities voluntarily participated in this study. The participants attended a two-day experimental protocol. On day 1 of the experimental protocol, the force–fascicle length relationships of the VL muscle were investigated by asking the participants to perform several maximal isometric voluntary knee extension contractions (MVC) on an isokinetic dynamometer. On day 2, VL fascicle length, EMG activity of the VL, vastus medialis (VM), rectus femoris (RF) and biceps femoris (BF), kinematics and ground reaction forces (GRF) were recorded during a jumping protocol. In the jumping protocol, the participants performed five SJs and five CMJs with their arms akimbo. The order of the two conditions was randomized and a rest interval of 2 min between the conditions and 30 s between the respective trials was included to provide regeneration for the participants. During all jumps, the maximum knee flexion angle was restricted to 90° (0° knee joint angle corresponded to neutral full extension). We restricted the knee joint angle to 90° by controlling the downward amplitude of the hip using haptic feedback. At the beginning of the experiment, we identified the position (i.e. height) of the hip where the knee joint angle was 90° for each participant using a motion capture system. We marked the identified height with an elastic rope fixed between two poles on the right and left side of the participants. After a short familiarization protocol, the participants were able to reproduce the targeted amplitude consistently. For the statistical analysis of all our data (i.e. fascicle length, EMG, kinematics and kinetics), we used the average values of the five trials of each condition.

### 2.2. Assessment of muscle intrinsic properties

Following a standardized warm-up including 5 min of ergometer cycling and five deep squats, the participants performed eight MVCs with their right leg in the range of 20° to 90° knee joint flexion angle in 10° intervals in a randomized order on an isokinetic dynamometer (Biodex 3, Biodex Medical Systems, Shirley, NY, USA). The knee joint angles were controlled and positioned at rest. However, because of the marked differences between rest and the plateau of the MVC due to soft tissue and dynamometer compliance [30], we measured the kinematics of the lower extremities during the MVCs using a Vicon motion capture system (v. 1.7.1., Vicon Motion Systems, Oxford, UK) integrating eight cameras (6 × Vicon F20, 2 × Vicon T20) operating at 250 Hz. Six reflective markers were placed on anatomical landmarks (anterior iliac spine, greater trochanter, lateral and medial femoral epicondyle and malleoli) and were recorded during the MVCs. The participants were instructed to push against the dynamometer continuously and hold the maximum moment for 2–3 s. To avoid fatigue, the MVCs were performed with at least 2 min rest between trials. The hip joint was set at a 85° position in order to reduce the contribution of the bi-articular RF to the resultant measured knee extension moments [27,28].

The resultant moments at the knee joint were calculated by means of inverse dynamics according to the methodology reported by Arampatzis *et al*. [30] to account (i) for the effect of the misalignment between knee joint axis and dynamometer axis and (ii) the effect of the gravitational forces. To evaluate the effect of the latter, the participants were instructed to relax their muscles while the leg was passively rotated around the knee joint from 0° to 90° knee joint flexion at 5° s^−1^. After three cycles, the kinematics of the leg and the moments were captured during knee extension to determine the moment due to the gravitational forces for each angular position. The contribution of the antagonistic moment of the hamstring muscles was considered by establishing a relationship between EMG amplitude and exerted moment of the hamstrings while working as agonist [31]. The EMG activity of the hamstrings and the corresponding moment were measured in a relaxed condition and two additional submaximal isometric knee flexion contractions of different intensity, according to the methodology reported by Mademli *et al*. [32]. EMG activity was measured synchronously with the kinematic data using a wireless EMG system (Myon m320RX, Myon AG, Baar, Switzerland) at a capture rate of 1000 Hz.

The force applied to the patellar tendon during the maximal isometric contractions was then calculated by dividing the knee joint moment by the angle-specific tendon moment arm. The patellar tendon moment arm at full knee extension was measured in a three-dimensional coordinate system as the perpendicular distance of the tendon's line of action to the rotation axis of the knee using magnetic resonance imaging (G-Scan, 0.25 T, 3D HYCE (GR) sequence, Esaote, Genova, Italy). The line of action of the patella tendon was defined as the line of best fit through the geometrical centres of the tendon cross-sectional areas between the caudal pole of the patella and the initial insertion at the tibial tuberosity, which were obtained from the segmentation of transverse plane magnetic resonance images. The corresponding axis of rotation of the knee joint was determined by segmenting the lateral and medial femoral epicondyles in the sagittal magnetic resonance scans and connecting the centres of the respective best fitting circles according to Churchill *et al*. [33]. The tendon moment arm as a function of the knee joint angle was calculated by processing moment arm changes in relation to joint angle on the basis of the data by Herzog & Read [34].

**Figure 1.**
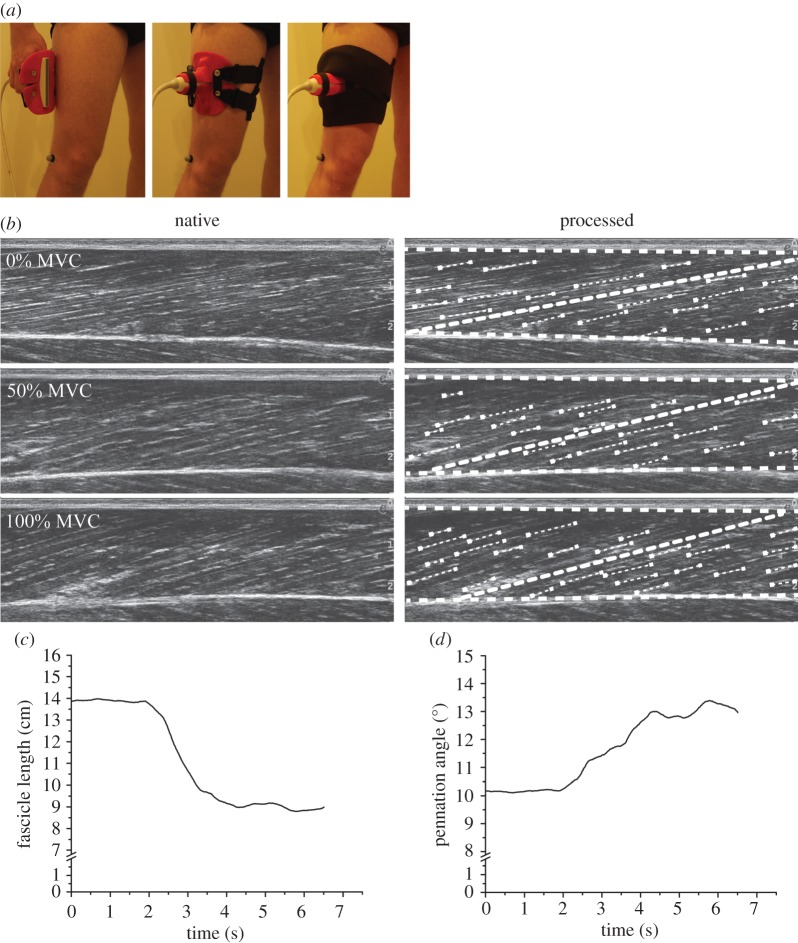
The ultrasound probe attached to the muscle belly by means of a customized neoprene elastic cast (*a*) and representative ultrasound images of vastus lateralis fascicles of one participant during rest, submaximal and maximal isometric voluntary knee extension contraction (MVC) at 50° knee joint angle (*b*). The last two graphs show the time course of fascicle length (*c*) and pennation angle (*d*) from rest to MVC.

**Figure 2.**
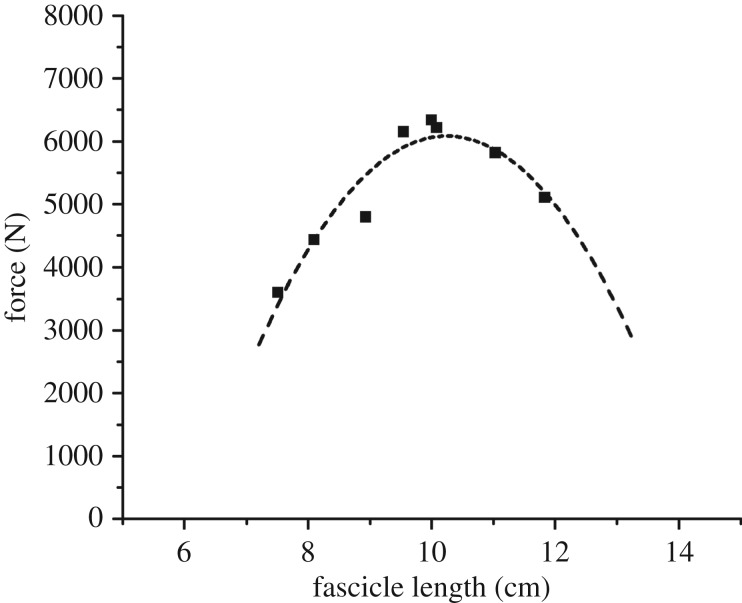
Representative experimentally determined force and vastus lateralis fascicle length (filled squares) during the maximal voluntary isometric knee extension contractions in different knee angles of one participant and the fitted curve (dashed line) using a second order polynomial.

Longitudinal ultrasound images of the VL were captured during the MVC trials in the eight knee joint angles using a 10 cm linear array probe (10 MHz, Esaote MyLab™60, Genova, Italy) at a capture frequency of 43 Hz. The ultrasound device and the Vicon motion capture system were synchronized with a trigger signal of 5 V, which was visible in both systems. The ultrasound probe was placed over the belly of the VL midway between trochanter major and epicondylus lateralis and was attached to the leg via elastic straps. The VL fascicle length was measured at rest and at the plateau of the MVC trials and was calculated as the average value based on the analysis of a 10 ultrasound images for each position. The recorded images were analysed using a custom-made tracking interface written in Matlab (v. R2011b, The Mathworks, Natick, USA). For each frame, the upper and deeper aponeuroses as well as visible features of multiple fascicles were manually digitized (figure 1). Visible fascicles were considered as valid when the length of the feature was more than or equal to 0.5 cm. The mean and standard deviation of the number of digitized visible fascicles for every video frame was 10 ± 7 (using a minimum of five fascicles per frame). A representative reference fascicle was then calculated on the basis of the orientation of the digitized fascicle portions. From the examined data of the MVCs (i.e. maximum force applied to the tendon as a function of measured VL fascicle length), we calculated a second order polynomial and determined the force–fascicle length curve for each participant (figure 2). The second order polynomial was used to determine the optimal length of the VL for force generation (*L*_0_) and the maximum muscle force applied to the tendon (*F*_max_). Specific muscle values taken from Miller *et al*. [29] were used (*a*_rel_ = 0.34 and *b*_rel_ = 4.03 s^−1^) to determine the maximal shortening velocity of the VL fascicle, which resulted in a calculated maximal VL shortening velocity (*V*_max_) of 11.85 *L*_0_ s^−1^. Subsequently, the force–velocity relationship of the VL fascicles was described following the classical Hill equation [11]. Owing to technical problems in the capturing of the reflective markers during the maximum isometric contractions we were not able to determine the force–fascicle length curve for two participants and, therefore, these participants were not included in the analysis of the operating length and velocity.

### 2.3. Measurement of ground reaction forces, kinematics, electromyographic activity and vastus lateralis fascicle length during jumping

During the jumping protocol, GRF and kinematic data were simultaneously captured using the Vicon system. Ground reactions forces of both legs were measured using two force plates (AMTI, BP400600-2000, 60 × 40 cm, Watertown, MA, USA) operating at a rate of 1000 Hz. The vertical take-off velocity of the centre of mass during the jumps was calculated through the integration of the vertical GRF over the time. Mechanical power applied to the centre of mass was determined as the product of vertical ground reaction force and vertical centre of mass velocity. The kinematic data were collected using the Vicon motion capture system operating at 250 Hz. Reflective markers were placed on anatomical landmarks on the right side of the body (fifth metatarsal head, lateral malleolus of the foot, lateral femoral epicondyle, greater trochanter, C7-vertebrae) to calculate joint kinematics. The three-dimensional coordinates of the markers were filtered using a second order low-pass Butterworth filter with a cut-off frequency of 6 Hz. The resultant ankle, knee and hip joint moments were calculated using inverse dynamics [35]. The mechanical power of each joint was calculated as the product of joint moment and joint angular velocity. Finally, the joint mechanical work during the propulsion phase of the jumps was calculated as the integral of the mechanical power over the time.

The EMG activity of VL, VM, RF and BF of the left leg was measured synchronously to the GRF and kinematics (sampling frequency 1000 Hz). The skin was shaved and cleaned with alcohol, and bipolar electrodes with a 2 cm inter-electrode distance were placed on the mid-portion of the muscle bellies. The EMG signal was then amplified (by the Myon wireless unit) and simultaneously transmitted to the Vicon system via a 16-channel A/D converter. Subsequently, the EMG signal was rectified and smoothed using a second order low-pass Butterworth filter with a cut-off frequency of 20 Hz. The filtered EMG data were then normalized to the averaged maximum value of each muscle attained during three maximal isometric voluntary knee extension and three knee flexion contractions at 60° knee joint angle that each participant had performed before the execution of the jumping protocol.

Ultrasonography was used to measure the VL fascicle length during the jumps. The ultrasound images were recorded using a 10 cm linear array probe at a capture frequency of 43 Hz and synchronized as described above. The ultrasound probe was carefully aligned to the VL muscle belly of the right leg using a custom-made plastic cast and the VL fascicle length was manually analysed afterwards using the procedure mentioned earlier (figure 1). The coefficient of variation of the fascicle angles of the single features that were used for the calculation of the representative reference fascicle in every image was 15% and 14% for the SJ and CMJ, respectively. The VL fascicle length behaviour was filtered using a second order low-pass Butterworth filter with a cut-off frequency of 6 Hz. The length changes of the VL muscle-tendon unit (MTU) were calculated as the product of the individual angle-specific patellar tendon lever arm and the change in knee joint angle [36]. The VL fascicle velocity and VL MTU velocities were then calculated as the first derivative of the fascicle and MTU lengths over the time.

### 2.4. Statistics

The differences in all investigated parameters between SJ and CMJ (i.e. fascicle length, fascicle-shortening velocity, force–length, force–velocity, power–velocity potentials, EMG, kinematics and kinetics) were checked using a paired *t*-test. The level of significance was set to *α* = 0.05.

## 3. Results

The experimentally assessed *L*_0_ of the VL muscle and *F*_max_ applied to the patellar tendon at *L*_0_ were 9.4 ± 1.1 cm and 4923 ± 929 N, respectively. Using the muscle-specific parameters *a*_rel_ = 0.34 and *b*_rel_ = 4.03 s^−1^ [29] the estimated *V*_max_ and the optimal shortening velocity (*V*_opt_) to generate maximal power (*P*_max_) of the VL were 111.4 ± 13.4 cm s^−1^ and 37.1 ± 4.5 cm s^−1^, respectively. Jump height and mean mechanical power applied to the centre of mass during the propulsion phase (i.e. start of the upward movement of the centre of mass until take-off) were significantly higher in the CMJ (*p* = 0.009 and *p* < 0.001) than in SJ but the maximal mechanical power did not show any significant differences (*p* = 0.663; table 1). The knee angle at the beginning of the propulsion phase (*p* = 0.316) as well the range of the knee angle extension (*p* = 0.886) during this phase did not differ between the two jumps (table 1). The time of the propulsion phase was significantly shorter in the CMJ (*p* < 0.001; table 1).

**Table 1.** Jump height, mean and maximal mechanical power applied to the centre of mass, knee angle at the beginning of the propulsion phase, range of knee angle extension and time of the propulsion phase during SJ and CMJ (average value ± s.d.).

parameter	SJ	CMJ
jump height (cm)	28.0 ± 3.6	29.5 ± 4.3^a^
mean mechanical power (Watt kg^−1^)	15.89 ± 3.11	25.00 ± 3.68^a^
maximal mechanical power (Watt kg^−1^)	46.78 ± 4.14	46.46 ± 4.84
knee angle at the beginning of the propulsion phase (°)	89.1 ± 6.6	90.2 ± 4.2
knee angle extension (°)	80.1 ± 5.8	79.9 ± 4.3
time of the propulsion phase (ms)	474 ± 82	302 ± 40^a^

^a^Statistically significant difference between SJ and CMJ (*p* < 0.05).

**Figure 3.**
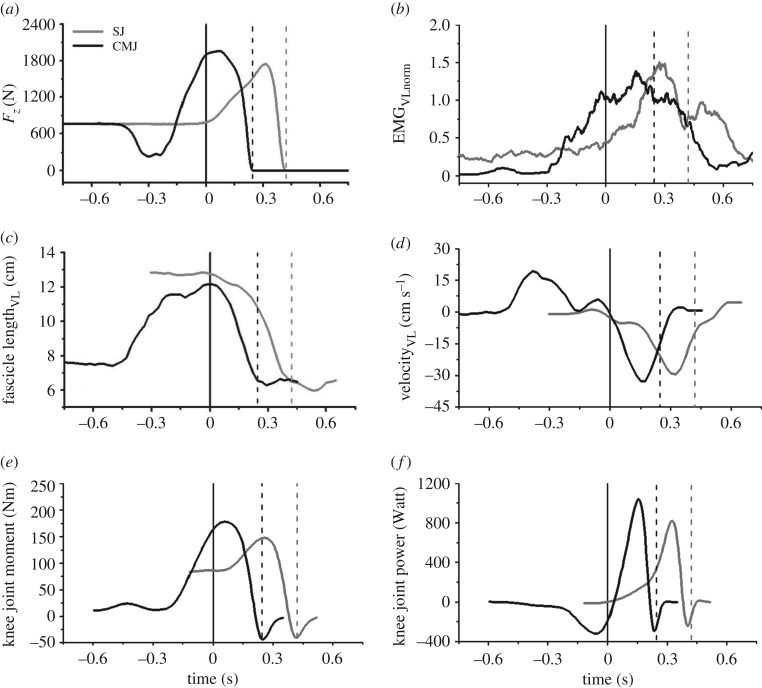
Vertical ground reaction forces (*a*), EMG activity of the vastus lateralis normalized to maximum isometric contraction (*b*), vastus lateralis fascicle length (*c*), vastus lateralis fascicle-shortening velocity (*d*), resultant knee joint moment (*e*) and knee joint mechanical power (*f*) during SJ and CMJ from a representative participant. Zero in the horizontal axis defines the beginning of the propulsion phase. The two additional vertical dashed lines define the take-off time points for SJ and CMJ. Note: resultant knee joint moments and knee joint mechanical power curves were calculated for the right leg.

The maximal resultant joint moments increased at the knee (*p* = 0.012) and hip (*p* < 0.001) joints in CMJ compared with SJ and did not show any statistically significant differences at the ankle joint (*p* = 0.443; table 2). Although the mean mechanical power was enhanced in all three joints in the CMJ (*p* < 0.001), the maximal joint mechanical power was only significantly higher at the hip joint (*p* = 0.007; table 2). The mechanical work during the propulsion phase increased at the knee (*p* = 0.036) and hip joint (*p* = 0.006), but not at the ankle joint (*p* = 0.338) in the CMJ compared with SJ (table 2). In the CMJ, the VL fascicles demonstrated a clear active lengthening during the downward phase of the jump. At the beginning of the propulsion phase, the fascicle length of the VL was shorter and the EMG activity of all investigated knee extensors showed greater values in the CMJ (*p* < 0.001; tables 3 and 4). The fascicles of the VL muscle shortened during the propulsion phase in both jumps and the EMG activity increased, reaching its maximum before take-off (figure 3). The shortening of the VL fascicles in the propulsion phase was larger in the SJ (*p* < 0.001), but the mean and the achieved maximum shortening velocity were larger in the CMJ (*p* < 0.001 and *p* = 0.005, respectively; table 3). The shortening of the VL MTU was larger compared with the VL fascicle (*p* < 0.001) but without any significant differences between the two jumps (*p* = 0.648). The mean and maximum shortening velocity of the VL MTU were higher in CMJ (*p* < 0.001, respectively). The ratio (CMJ to SJ) of the mean VL MTU velocity and VL fascicle velocity was significantly lower on the fascicle level (1.31 ± 0.25 versus 1.58 ± 0.31, *p* < 0.001), indicating a smaller increase in shortening velocity from SJ to CMJ in fascicles compared with the MTU. All investigated leg muscles demonstrated a significantly higher average EMG activity during the propulsion phase in CMJ compared with SJ (*p* < 0.001; table 4).

**Table 2.** Maximal resultant joint moments, mean and maximal joint mechanical power and joint mechanical work during the propulsion phase of SJ and CMJ (average value ± s.d.). Note: the values represent only the right leg.

parameter	SJ	CMJ
maximal ankle joint moment (Nm kg^−1^)	1.43 ± 0.20	1.47 ± 0.30
maximal knee joint moment (Nm kg^−1^)	1.47 ± 0.22	1.60 ± 0.32^a^
maximal hip joint moment (Nm kg^−1^)	1.78 ± 0.32	2.18 ± 0.43^a^
mean ankle joint mechanical power (Watt kg^−1^)	2.03 ± 0.53	3.24 ± 1.12^a^
mean knee joint mechanical power (Watt kg^−1^)	1.99 ± 0.53	3.31 ± 0.81^a^
mean hip joint mechanical power (Watt kg^−1^)	2.81 ± 0.89	4.72 ± 0.96^a^
maximal ankle joint mechanical power (Watt kg^−1^)	9.94 ± 1.44	9.72 ± 1.74
maximal knee joint mechanical power (Watt kg^−1^)	8.62 ± 1.43	8.86 ± 1.72
maximal hip joint mechanical power (Watt kg^−1^)	7.92 ± 1.84	8.40 ± 1.54^a^
mechanical work at the ankle joint (J kg^−1^)	0.90 ± 0.15	0.93 ± 0.19
mechanical work at the knee joint (J kg^−1^)	0.89 ± 0.15	0.97 ± 0.16^a^
mechanical work at the hip joint (J kg^−1^)	1.28 ± 0.38	1.42 ± 0.33^a^

^a^Statistically significant difference between SJ and CMJ (*p* < 0.05).

**Table 3.** M. vastus lateralis fascicle length at the beginning and the end of the propulsion phase, vastus lateralis fascicle lengthening during the downward phase of the CMJ, fascicle and muscle-tendon unit shortening, mean and maximum shortening velocity of the vastus lateralis fascicles and muscle-tendon unit during the propulsion phase in SJ and CMJ (average value ± s.d.).

parameter	SJ	CMJ
fascicle length at the beginning of the propulsion phase (cm)	13.2 ± 1.1	12.1 ± 1.1^a^
fascicle length at the end of the propulsion phase (cm)	6.8 ± 0.7	6.8 ± 0.7
fascicle lengthening (cm)	—	4.5 ± 1.5
fascicle shortening (cm)	6.4 ± 1.3	5.3 ± 1.2^a^
mean fascicle-shortening velocity (cm s^−1^)	13.8 ± 3.3	17.6 ± 3.9^a^
maximum fascicle-shortening velocity (cm s^−1^)	35.4 ± 9.3	41.3 ± 8.5^a^
muscle-tendon unit shortening (cm)	7.9 ± 4.4	7.8 ± 4.2
mean muscle-tendon unit shortening velocity (cm s^−1^)	16.9 ± 3.1	26.1 ± 3.5^a^
maximum muscle-tendon unit shortening velocity (cm s^−1^)	64.3 ± 3.5	67.1 ± 3.9^a^

^a^Statistically significant difference between SJ and CMJ (*p* < 0.05).

**Table 4.** EMG activity normalized to maximum isometric contraction at the beginning of the propulsion phase (begin) and mean EMG activity during the propulsion phase (propulsion) in the vastus lateralis (VL), vastus medialis (VM), rectus femoris (RF) and biceps femoris (BF) muscles during SJ and CMJ (average value ± s.d.).

parameter	SJ	CMJ
EMG activity of VL_begin_ (%)	55.4 ± 24.9	102.4 ± 35.5^a^
EMG activity of VM_begin_ (%)	65.1 ± 36.3	131.6 ± 73.3^a^
EMG activity of RF_begin_ (%)	59.7 ± 30.2	103.6 ± 50.1^a^
EMG activity of BF_begin_ (%)	15.0 ± 15.9	42.7 ± 29.1^a^
mean EMG activity of VL_propulsion_ (%)	107.6 ± 43.9	123.4 ± 48.6^a^
mean EMG activity of VM_propulsion_ (%)	124.2 ± 56.4	140.5 ± 62.4^a^
mean EMG activity of RF_propulsion_ (%)	119.3 ± 40.8	143.1 ± 56.2^a^
mean EMG activity of BF_propulsion_ (%)	37.8 ± 36.3	45.0 ± 25.4^a^

^a^Statistically significant difference between SJ and CMJ (*p* < 0.05).

**Figure 4.**
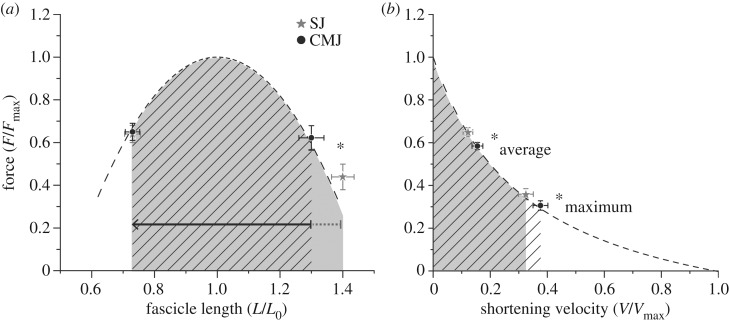
Operating length (*a*) and velocity (*b*) of vastus lateralis (VL) fascicles onto the normalized force–length and force–velocity curves during SJ and CMJ (mean values ± s.e.). Force was normalized to the maximum force obtained during the maximal isometric contractions, fascicle length to the experimentally determined optimal fascicle length and fascicle-shortening velocity to the estimated maximal shortening velocity. The vertical axes represent the force potential (fraction of VL maximum force); the force–length potential was determined at the beginning of the propulsion phase and take-off (fascicle shortening is indicated by the arrows); the force–velocity potential was determined for the mean vastus lateralis fascicle-shortening velocity (*average*) and *maximum* shortening velocity during the propulsion phase. The marked areas under the curve indicate the range of the operating length and velocity of the VL fascicles during the propulsion phase of the SJ (solid) and CMJ (hatched). Asterisks indicate statistically significant difference in force–length and force–velocity potential between SJ and CMJ (*p* < 0.05).

**Figure 5.**
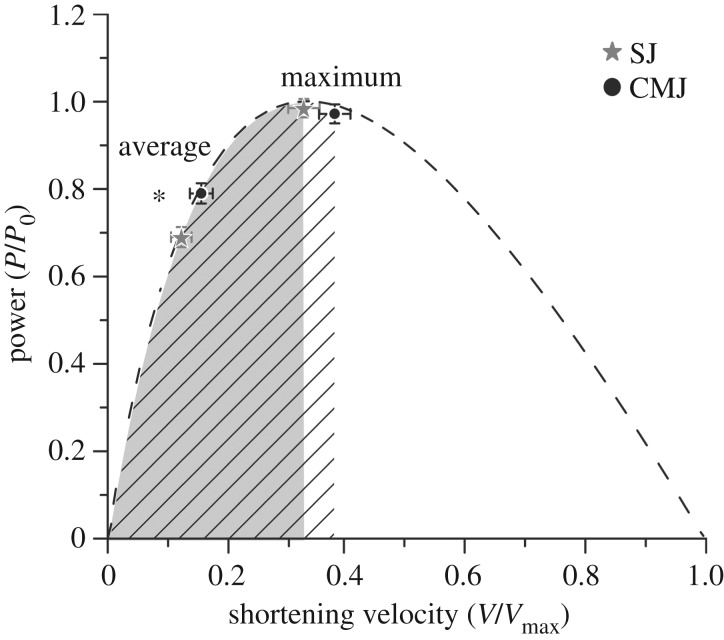
Operating velocity of the vastus lateralis (VL) fascicles onto the normalized power–velocity curve during SJ and CMJ (mean values ± s.e.). Power was normalized to the maximum power predicted from the force–velocity curve and fascicle-shortening velocity to the estimated maximal shortening velocity. The vertical axis represents the power potential (fraction of VL maximum power). *average* is the mean VL fascicle-shortening velocity and corresponding power potential during the propulsion phase. *maximum* is the maximum shortening velocity and corresponding power potential. The areas under the curve indicate the range of the operating velocity of the VL fascicles during the propulsion phase of the SJ (solid) and CMJ (hatched). Asterisks indicate statistically significant difference in the power–velocity potential between SJ and CMJ (*p* < 0.05).

At the beginning of the propulsion phase, the VL fascicles operated on the descending part of the force–length curve in both jumps; however, in the CMJ fascicle length was significantly closer to *L*_0_ (relative values to *L*_0_ for SJ 1.40 ± 0.14 and for CMJ 1.30 ± 0.09, *p* < 0.001; figure 4). During the push-off the fascicles passed the optimal length and continued to operate on the ascending part of the force–length curve until take-off in both jumps (relative values to *L*_0_ at take-off for SJ 0.73 ± 0.1 and for CMJ 0.73 ± 0.09; figure 4). In summary, the VL fascicles operated closer to the *L*_0_ in the CMJ than during the SJ in the propulsion phase, which resulted in a significantly larger average force–length potential (*p* = 0.001; table 5). The larger mean shortening velocity during the propulsion phase in the CMJ, on the other hand, resulted in a significantly lower force–velocity potential compared with the SJ (*p* < 0.001; table 5 and figure 4). The VL fascicles operated on the ascending part of the power–velocity curve in both jumps during the whole propulsion phase (figure 5) and the maximum fascicle-shortening velocity during the jumps was very close to the *V*_opt_ without any differences in maximum power–velocity potential between the two jumps (*p* = 0.564; table 5 and figure 5). However, the mean shortening velocity in the CMJ was closer to *V*_opt_, which resulted in a significantly higher average power–velocity potential during the propulsion phase compared with the SJ (*p* < 0.001; table 5 and figure 5).

**Table 5.** Fraction of vastus lateralis (VL) maximum force according to the force–length (force–length potential) and force–velocity (force–velocity potential) relationships as well as fraction of the vastus lateralis maximum power according to the power–velocity (power–velocity potential) relationship during SJ and CMJ. *Mean*: average values during the propulsion phase, *border*: values at maximum shortening velocity obtained during the jumps (average value ± s.d.).

parameter	SJ	CMJ
force–length potential of VL_mean_	0.77 ± 0.07	0.82 ± 0.07^a^
force–velocity potential of VL_mean_	0.65 ± 0.06	0.58 ± 0.06^a^
force–velocity potential of VL_border_	0.36 ± 0.10	0.30 ± 0.08^a^
power–velocity potential of VL_mean_	0.69 ± 0.10	0.79 ± 0.09^a^
power–velocity potential of VL_border_	0.96 ± 0.05	0.96 ± 0.04

^a^Statistically significant difference between SJ and CMJ (*p* < 0.05).

## 4. Discussion

The current investigation, based on an *in vivo* assessment of the human VL fascicle length and shortening velocity during SJ and CMJ, provides for the first time evidence that both the muscle intrinsic force–length and power–velocity relationships as well as muscle activation contribute to the marked differences in the mean mechanical power output (56%) between the two jumps. We found that the mean VL shortening velocity during the propulsion phase in the CMJ was near to the optimal shortening velocity for power production and closer to the optimum compared with the SJ, providing a 15% greater power potential due to the power–velocity relationship. The mean EMG activity of the VL muscle was also approximately 15% higher during the CMJ, indicating a greater muscle activation level during the push-off phase. The force–length and force–velocity potentials showed a different constellation during the jumps. While the force–length potential was higher (7%) in the CMJ, the force–velocity potential was higher (11%) in the SJ, indicating a counteracting effect during the jumps. The relatively moderate, considering the differences in mean mechanical power, increase in jump height (5%) in the CMJ can therefore be explained by the on average larger muscle activation during the propulsion phase. In the CMJ the maximum resultant moment, mean mechanical power and mechanical work at the knee joint during the propulsion phase increased by 9%, 66% and 9%, respectively, compared with the SJ. These values indicate that, although muscles acting at the ankle and hip joints contribute to the performance-enhancement (i.e. jump height and mean mechanical power) in CMJ, the knee extensors are important contributors to the found differences. The findings confirmed our hypotheses and demonstrate that the intrinsic mechanical mechanisms of the VL muscle (i.e. force–length and power–velocity potentials) are relevant contributors to the increased mean mechanical power output in the CMJ compared with the SJ.

We conducted simultaneous measurements of VL fascicle length changes and activation patterns during SJ and CMJ. The experimentally assessed optimal length of the VL fascicles was 9.4 ± 1.1 cm and, thus, very close to the VL optimal fibre length determined by measurements of sarcomere lengths (i.e. 9.9 ± 1.7 cm; [37]). In the downward phase of the CMJ, the fascicles of the VL lengthened on average 47% of the *L*_0_, starting from the ascending part and finishing on the descending part of the force–length curve. During this phase, all investigated muscles of the quadriceps femoris (VL, VM and RF) were active, but their maximal EMG activity was achieved during the shortening phase. In both jumps, the initiation of the push-off phase was performed at the same knee angle (i.e. same length of the VL MTU), but the fascicle length of the VL was on average 1 cm shorter in the CMJ compared with the SJ. This difference can be explained by tendon compliance and the higher active state of the VL muscle in the CMJ. Experimental data during maximal isometric contractions reported elongations of the VL tendon of 1.5 cm corresponding to strain values of approximately 8% [38]. Musculoskeletal models [4] predicted a significantly higher muscle force of the vastii (a factor of 2.7) at the beginning of the upward phase in the CMJ compared with SJ, which renders the observed 1 cm difference in the VL fascicle length in this study to be realistic. The consequence was that, at the same length of the MTU, the force generating potential of the VL muscle was higher in the CMJ due to the force–length relationship. This means that at the time of the initiation of the push-off phase both a higher activation level and a higher force–length potential of the VL muscle promoted muscle force and power generation in the CMJ compared with SJ.

During the propulsion phase the VL fascicles first operated toward optimal length for force generation in both jumps and then in the ascending part of the force–length curve showing a substantial shortening. The shortening of VL fascicles in the SJ was larger (68% of *L*_0_) than in the CMJ (57% of *L*_0_). The extensive shortening of the VL fascicles in combination with the high EMG activity during the push-off phase is indicative of a substantial mechanical work production during the jumps. Despite a larger shortening of the VL fascicles in the SJ, the maximal and mean shortening velocity were greater in CMJ (16% and 28%, respectively). This indicates that as the VL muscle underwent an active shortening and performed mechanical work, it operated on a more favourable portion of the force–length curve and on a more disadvantageous portion of the force–velocity curve in the CMJ compared with the SJ, suggesting a reciprocal effect of force–length and force–velocity potentials for muscle force generation. The increase in shortening velocity in the CMJ is to some degree attenuated by tendon compliance. The lower ratio (CMJ to SJ) of the VL fascicle mean velocity compared with the VL MTU mean velocity shows that the elongation of the tendon prevents a similar increase in VL fascicle-shortening velocity as in the MTU during the CMJ and that the facilitation of the force–velocity potential due to tendon compliance is greater during the CMJ compared with the SJ. We conclude from these results that during the push-off phase the increased activity of the VL muscle in the CMJ and not an advantageous muscle intrinsic force–length–velocity relationship facilitates muscle force generation and can explain the moderate differences in jump height between SJ and CMJ. Therewith, our experimental data confirm predictions from modelling studies reporting that the higher activation of the extensor muscles is the responsible mechanism for the higher jumping height in CMJ [5].

The shortening velocity of the VL fascicles ranged from the ascending part of the power–velocity curve to the optimal velocity for power generation in both jumps. The maximal VL fascicle-shortening velocities achieved during the jumps were close to the plateau of the power–velocity curve (33% of *V*_max_ for the SJ and 38% of *V*_max_ for the CMJ) without any significant differences in the maximum power potential between the two jumps. The mean velocity values of the VL fascicles relative to the *V*_max_ during the push-off phase were 16% for the CMJ and 12% for the SJ. The consequence of this behaviour (i.e. operation of the VL fascicle-shortening velocity in the ascending part of the power–velocity curve) during the propulsion phase was a more favourable average power potential in the CMJ. The larger mean shortening velocity of the VL was closer to the plateau of the power-velocity curve in CMJ and resulted in a 15% greater average power potential in the push-off phase of the CMJ. Taken into consideration that also the force–length potential and the EMG activity of the VL during the propulsion phase were higher in the CMJ (7 and 15%, respectively) and that both a more favourable force–length potential and activation enhance the power output of a muscle, we provide evidence for a cumulative effect of three different mechanisms for an advantaged power production in the CMJ. Consequently, our results lend strong support to the important role of intrinsic mechanical mechanisms of the VL muscle (i.e. force–length and power–velocity relationships) as well as muscle activation level regarding the clear increase in mean mechanical power applied to the centre of mass in the CMJ compared with the SJ.

The mechanical power applied to the centre of mass during the propulsion phase can be affected by the energy storage and return in the series elastic elements of the muscle-tendon units in a catapult-like manner, causing power amplification [8,39]. Although experimental [21,22] as well as simulation studies [4] reported similar storage and utilization of elastic strain energy during SJ and CMJ, in the beginning of the push-off phase the tendon strain energy is greater in the CMJ. This is happening due to a transfer of gravitational potential energy to the tendons during the downward phase [4]. The existing elastic strain energy in the system can affect the time course of mechanical power output, because the total power output depends on the release of stored energy and muscular power production and our data (i.e. the CMJ/SJ ratios of fascicle and MTU shortening velocity) suggests that elastic strain energy contributed to the enhancement of the mean mechanical power observed in the CMJ. It is well known that following active muscle stretching or shortening, history-dependent phenomena of force production such as force enhancement and force depression also can affect muscle force and power production [40]. Although McGowan *et al*. [41], using a simulation model, found that CMJ performance was very similar with and without force enhancement/depression phenomena of muscle contraction, providing evidence that history-dependent effects during CMJ were compensated by changes in muscle activation, it cannot be excluded that force depression phenomena during SJ may affect the muscle's ability to generate force. However, this possibility does not reduce the importance of our findings that intrinsic mechanisms of the VL muscle to generate force and power contribute to the higher mean mechanical power output during the CMJ. Considering that the contractile element of the VL is a dominant contributor to energy and power supplied to the skeleton during SJ and CMJ [4,7], our conclusion regarding the importance of intrinsic mechanisms of VL as crucial explanatory factors for the clear differences in the mean mechanical power output between the two jumps, can be further supported.

In this study, we measured the VL fascicle length and calculated the shortening velocity using an ultrasound device with a linear array probe that is longer (i.e. 10 cm) than most of the commonly used ultrasound systems. The 10 cm probe is very important for the accurate measurement of long fascicles as in the VL, because almost the whole fascicle is visible in the image. The used capture frequency of 43 Hz, however, was lower compared with the capture frequency of the Vicon system (250 Hz for kinematic data and 1000 Hz for analogue data), which may introduce some bias in the measurement of the peak VL shortening velocity. Our main conclusions, however, are based on the mean shortening velocity during the propulsion phase of the two jumps. The average time of the push-off phase was 474 and 302 ms for the SJ and CMJ, respectively. This means that the calculation of the mean shortening velocity was based on, in average, 20 ultrasound images for the SJ and 13 for the CMJ, which in the authors' opinion is enough to obtain representative values. Of course ultrasound provides only a two-dimensional image, therefore the fascicle length will be underestimated in the case that the digitized fascicles do not lie in the image plane. Studies investigating the validity of the ultrasound-based determination of fascicle length reported an acceptable validity for VL and vastus intermedius [42] as well as for GM [43]. In general, the ultrasound-based methodology shows a systematic underestimation of the VL fascicle length with average differences of approximately 6% [42].

In our study, we examined the force–length relationship of VL fascicles and we assessed the optimal VL fascicle length and the maximum voluntary isometric force in all participants experimentally. For the assessment of the maximum shortening velocity, we used muscle-specific parameters from Miller *et al*. [29], which predicted a maximum VL shortening velocity of 11.85 *L*_0_ s^−1^. As the optimal shortening velocity for maximal power production is a fraction of *V*_max_ (approx. 33%), our prediction determined the range of the ascending part of the power–velocity relationship, which in turn influenced our attribution of the measured mean shortening velocity of the VL during SJ and CMJ to the ascending part. We measured mean VL fascicle-shortening velocities during SJ and CMJ of 1.44 *L*_0_ s^−1^ and 1.84 *L*_0_ s^−1^, respectively. In the case that the VL optimal shortening velocity for power production would be higher than 1.84 *L*_0_ s^−1^, the measured mean shortening velocity of VL in SJ (1.44 *L*_0_ s^−1^) would be in the ascending part of the power–velocity relationship and the power potential in SJ would be lower than in the CMJ. This means that the lowest threshold of the VL *V*_max_ for this assumption to hold true would be a *V*_max_ of 5.57 *L*_0_ s^−1^, where the corresponding optimal shortening velocity would be 1.84 *L*_0_ s^−1^, assuming an approximate peak power at 33% of *V*_max_ [11]. A lower *V*_max_ than this threshold seems to be unlikely for the VL, a muscle with a proportion of fast-twitch fibres between 54% and 59% [44–46]. Reports from Ranatunga [47] predict maximum shortening velocities for the slow-twitch rat soleus of 7.71 *L*_0_ s^−1^ and for the fast-twitch extensor digitorum longus of 14.15 *L*_0_ s^−1^ at 37° Celsius (human *in vivo* temperature). A recent study by Fontana *et al*. [48] investigated the force–velocity relationship of the human VL *in vivo* and their data predict a maximum shortening velocity of approximately 6.0 *L*_0_ s^−1^, which is only slightly higher than our calculated threshold of 5.57 *L*_0_ s^−1^. However, it needs to be considered that both the submaximal activation levels (i.e. approximately 70%) during the isokinetic contractions [49] as well as not taking into account hamstring co-activation in the estimation of fascicle forces (i.e. approximately 20%, [50]) probably affected the predicted shape of the force–velocity relationship and led to an underestimation of maximum shortening velocity in the study by Fontana and co-workers [48]. Furthermore, Finni *et al*. [51] investigated the patellar tendon force–VL fascicle-shortening velocity using the optic fibre methodology and found peak VL shortening velocity of 20 cm s^−1^ at a patellar tendon force of 57% of the isometric maximum. Using these values (shortening velocity of 20 cm s^−1^ at 57% of isometric maximum) the Hill equation predicts a maximum shortening velocity for the VL muscle of 124 cm s^−1^ (i.e. 6.2 times the velocity at 57% maximum force; figure 4) corresponding to a 13.2 *L*_0_ s^−1^ velocity. We believe that the reports mentioned above provide convincing physiological evidence that the VL maximum shortening velocity is significantly higher than 5.57 *L*_0_ s^−1^.

We assessed the force–length curve during maximal isometric contractions at different knee joint angles and, using this relationship, we calculated the force–length potential of the VL muscle. There is evidence that the force–length curve depends on muscle activation (i.e. optimum length increases with decreased activation; [52]), which demonstrates that using force–length curves from maximal activated muscles to understand neurophysiological mechanisms during daily life tasks with submaximal activated muscles has limitations. However, we measured an average EMG activity in the propulsion phase during the SJ and CMJ of 107% and 123% of the maximum isometric EMG activity, which indicates quite maximal muscle activity during the jumps and suggests that effects of activation-dependency on our interpretations are negligible. Finally, we evaluated the VL muscle as a representative of the quadriceps muscles in our experiment. We found a significant increase in mean mechanical power also at the ankle (60%) and hip joints (67%) in the CMJ. Further the contribution of the ankle, knee and hip joint to the total mechanical work within the lower extremities was 29%, 29% and 42% for the SJ, respectively, and 28%, 29% and 43% for the CMJ. This means that other muscles in the lower extremities also contribute to the performance enhancement during the CMJ, which partly can be investigated in a similar manner using the proposed methodology.

## 5. Conclusion

Jumping height and mean mechanical power applied to the centre of mass during SJ and CMJ can be affected by the operating length and velocity of the quadriceps vastii muscles, which are responsible for the large differences in force and energy production between the two jumps. Therefore, it is important to understand the potential of proximal muscles for force and power production in human jumping. In the current study, we focused on the VL fascicle length changes and activation patterns to understand the involvement of neuro-mechanical muscle intrinsic mechanisms on human jumping and to provide new knowledge regarding the different performance output of SJ and CMJ. We found that VL fascicles operate on a more advantageous portion on the force–length and power–velocity curve during CMJ and on a more advantageous portion of the force–velocity curve during SJ. Finally, the findings of this study lead us to conclude that three important mechanisms for increased muscle power production were favourable in CMJ compared with SJ. During the propulsion phase of the CMJ (i) the fascicles of the VL muscle operate at lengths near the plateau of the force–length curve, (ii) the fascicles operate at shortening velocities near to the optimal velocity for power production and (iii) the VL muscle is subjected to a higher activation level than during the SJ.

## Data accessibility

Supporting data are available at (http://dx.doi.org/10.5061/dryad.sd632) [53].
